# Characterization of Key Aroma Compounds and Construction of Flavor Base Module of Chinese Sweet Oranges

**DOI:** 10.3390/molecules24132384

**Published:** 2019-06-27

**Authors:** Mengzhu Shui, Tao Feng, Yanzun Tong, Haining Zhuang, Chihkang Lo, Hongfeng Sun, Ling Chen, Shiqing Song

**Affiliations:** 1 School of Perfume and Aroma Technology, Shanghai Institute of Technology, No.100 Haiquan Road, Shanghai 201418, China; 2 Institute of Edible Fungi, Shanghai Academy of Agricultural Sciences, Key Laboratory of Edible Fungi Resources and Utilization (South), Ministry of Agriculture, National Engineering Research Center of Edible Fungi, 1000 Jinqi Road, Shanghai 201403, China; 3 Shanghai Kangshi Food Technology Co., Ltd., No.1978 Lianhua Road, Shanghai 201103, China

**Keywords:** Chinese sweet orange, flavor, key aroma compounds, notes, flavor base module

## Abstract

Sweet orange flavor, with its refreshing, joyful and attractive aroma, is favored by the majority of consumers all over the world. However, the industry terminology between flavorists for flavor evaluation is a bit vague and not intuitive for customers. Therefore, the study focused on analysis of sweet orange aroma and establishment of base module of orange flavor. The approach to the research involves screening key aroma compounds, identifying the attributes aroma and building base module of sweet orange. The notes of sweet orange flavor were determined by GC-O olfaction and sensory evaluation. 25 key aroma compounds with OAV ≥ 1 were screened and divided into eight notes: citrus, fruity, fresh, green, peely, woody, fatty, floral. Partial least squares regression (PLSR) was used to further verify the corresponding relationship between the volatile substances and notes. Terpenes, esters, aldehydes and alcohols compounds can provide these notes. Based on the notes, 8 base modules of sweet orange were built by selecting and matching aroma ingredients. Through this study, beginners could be trained according to the 8 notes of base modules and flavorists can engage in dialogue with different raw material sourcing teams or providers.

## 1. Introduction

Oranges have become more and more popular in recent years due to their preferable flavors and the nutrient value. The annual world production of oranges has been estimated to be 47.8 million tons [1]. Oranges are produced in more than 140 countries around the word, at latitudes between approximately 40° N and 40° S [2]; the main orange-producing countries are USA (California, Florida and Arizona), Mexico, Brazil, Spain, Italy, Israel, Australia, South Africa, Japan and China [3,4]. According to previous reports [5,6], oranges are rich in a variety of metabolically active substances, such as vitamin C, carotenoids, flavonoid, coumarin and phenolic compounds; these components are very important for human health and provide protection against harmful free radicals. Sweet oranges are mostly consumed as fresh fruit, juice, canned orange segments, and wine, and even the peel can be used in essential oils, which are widely used in the fragrance and flavor industries [7,8].

Aroma is an important character of sweet oranges and a key indicator for evaluating sweet oranges quality. Up until now, approximately 80 aroma compounds have been determined in orange juices, of which 15 volatile components presented odor activity values (OAVs) greater than 1, with dl-limonene, nootkatone and linalool being those with the highest OAVs in both cultivars [9,10]. To better understand the diversity and interrelationship of different sweet orange cultivars, morphological and RAPD markers have been applied in classification [11,12,13,14]. Volatiles of 4 sweet orange oils were characterized by descriptive sensory analysis and principal component analysis. Results showed that the important sensory factors including green, fatty, floral, woody, peely [15]. Sensory attributes of the orange pulp (from Brazil and Florida, U.S.A.) were orange, orange peel, pine-like, fresh, overripe, and oxidized according to sensory sniffing [16].

As one of the origin areas of sweet oranges, China is rich in citrus germplasm resources, including many wild and cultivated species [17]. Zigui, located in the Three Gorges Valley of the Yangtze River, is a famous “winter warm center” in China and the most suitable area for orange cultivation; Meishan sweet orange is rooted in a deep alluvial plain with fertile slightly acidic soil. The Minjiang River and the Qingyijiang River provide a clear water source for it, which is the best environment for sweet orange growth; Jiangyong sweet orange was well known in the Ming and Qing Dynasties because of its strong flavor and sweet taste; Yaoxiang has a subtropical monsoon climate with annual rainfall of 1200–1800 mm, and annual sunshine hours of more than 1400 h, which are very beneficial to the quality of sweet oranges; Qingyang is located between 35° N 14′28″ and 37° N 9′13″ and is 1100–1600 m above sea level. It belongs to the dominant area for sweet oranges in the northwest loess plateau. While each type of sweet orange has its own flavor signature, the flavor types may greatly vary within the different species, and the volatile compounds of sweet orange have also been traditionally monitored.

The immense popularity of citrus flavor is evidenced by the fact that orange is one of the top flavors sold world-wide by the commercial flavor industry [18,19]. Therefore, consumption of sweet orange flavor has been applied to beverages, baking, candy, ice cream and other food processing. Especially in beverages, sweet orange flavors are used to mask off-notes caused by vitamins, minerals and other nutraceutical components [20]. However, for sweet orange flavor, there exist many problems in practical applications, such as flavor in the processing and storage easily reacts with other components; causes undesirable aromas; and reduces the stability of the products.

There is a lack of consistent description between flavor suppliers and customers regarding aroma quality and optimization of sweet orange flavor. In addition, flavorists have their own language system for flavor. To get rid of these misunderstandings and rely on the same mode of description, a profile analysis of complicated flavor composition is carried out in this paper. Therefore, the aims of the present study are to (1) identify the key aroma compounds of five kinds of sweet oranges with different growing places using multivariate analysis, (2) use PLSR to confirm the relationship between the volatile compounds and notes, (3) construct the outline diagram of notes, based on which the composition and proportion of sweet orange aroma can be seen intuitively, and (4) establish a sweet orange flavor base module to evaluate the aroma of sweet orange flavors, according to the different aroma note categories. Through the establishment of the flavor base module in sweet orange, customers can intuitively evaluate and give feedback on the sweet orange flavor provided by flavor suppliers, which can create more competitive flavor products.

## 2. Results and Discussion

### 2.1. Volatile Compounds of Sweet Orange Determined by GC-MS

A total of 49 volatile compounds were identified and quantified after the GC-MS analysis of orange juice samples, by identification of retention index (RI), odor descriptors with authentic standard, as shown in Table 1. There were 35, 31, 30, 28 and 29 volatile compounds corresponding to the different orange samples, respectively. Hydrocarbons were predominant in the headspace gas of orange juice, followed by esters, aldehydes, alcohols ketones and acids. It has been reported that sweet oranges displayed higher levels of monoterpenes, especially d-limonene [18]. Our results generally agreed with that data; limonene was the most abundant monoterpene hydrocarbon tested in this study, followed by o-cymene, p-cymene, myrcene and valencene. Limonene was the main constituent of all samples. ZG samples contained the highest percentage of limonene (24.4 mg/L). ZG and MS samples contained a high percentage o-Cymene and 4-isopropyltoluene, and a high percentage of myrcene was present in ZG and YX samples. Of the esters, a large amount of ethyl butyrate was present in QY. In addition, the ZG and QY samples contained a large amount of acetaldehyde; additionally, ZG and MS samples also contained a large amount of linalool. Other studies revealed that linalool was the most abundant alcohols in Archi citrus juice [21].

### 2.2. Characterizations of Odor-Active Compounds by GC-O Analysis

The results of the olfactometry analysis are summarized in Table 2. Ethyl butyrate, myrcene, limonene, p-cymene, and linalool were the major odor-active compounds according to their high aroma intensity; these compounds were generally associated with pineapple, floral, woody, lemon, green and leaf aromas in the sensory descriptions of the panelists (Table 2). Among the hydrocarbon compounds, limonene (7.5–7.8) was present with the highest aroma intensity in all five samples, having a typical lemon aroma. Meanwhile, α-pinene (1.6–2.8) and myrcene (1.3–6.3) were also present in all samples. 4-isopropyltoluene (1.6–7.5) had the highest aroma intensity in ZG, MS, and JY, and was an important volatile compound contributing to the green and herbal aroma in orange samples. In addition to hydrocarbons, esters were another important class of odor-active compounds, with apple, pineapple, pear, etc., fruity aromas. Ethyl butyrate (5.3–7.5) possessed the highest aroma intensity among these compounds. Ethyl isobutyrate (1.7), ethyl isovalerate (2.5) and propyl butyrate (1.9) were only detected in QY.

### 2.3. Calculating OAV Values of Volatile Compounds in Sweet Orange and Percentage of Notes Contribution

As is shown in Table 3, there were 13, 16, 10, 12 and 16 compounds (OAVs ≥ 1) corresponding to the ZG, MS, JY, YX and QY samples that were considered to contribute significantly to the aroma of the samples. In this study, ethyl butyrate (5–233), myrcene (28–90), limonene (96–122), 4–isopropyltoluene (46–146), linalool (10–136) and decanal (22–66) had relatively higher OAVs, and were powerful aroma compounds in the five sweet orange samples. According to the GC-O olfaction of sensory evaluation panelists and the scores of notes in five sweet orange samples, 25 key aroma substances with OAVs ≥ 1 were classified into 8 notes. The fresh note of sweet orange was all derived from acetaldehyde; hexanol and hexanal had a strong aroma of vegetables and grass, so they were classified as green note; all esters contributed to the characteristic fruits note; all terpene compounds, including limonene and terpinene, provided the characteristic aroma for the citrus note; linalool and hedion had the fragrance of roses and jasmine, which were classified as the note of flowers; decanal with long chain carbon had the peculiar aroma of citrus peel; trans-caryophyllene and germacrene had distinct aroma of wood and herbs, which were attributed to the woody note.

**Figure 1:**
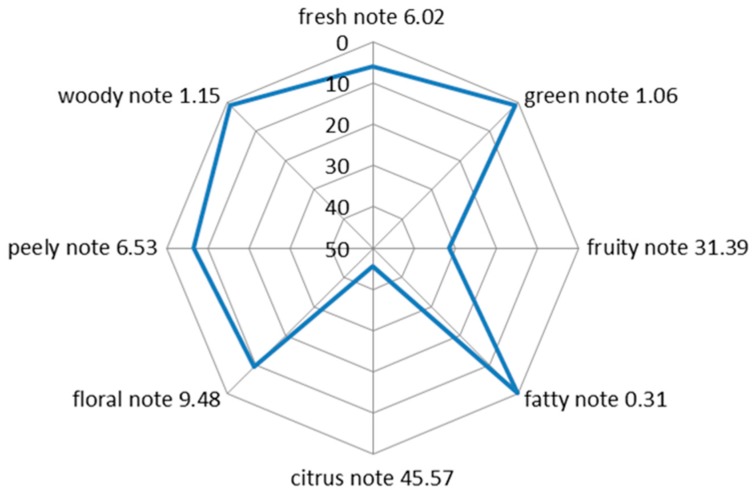
The notes contour of sweet orange flavor (the percentage of aroma notes was larger in the inner concave, but smaller in the outer convex and sharp one).

We can intuitively see the percentage contribution of each note in Figure 1. The percentage contribution of citrus note was 45.57%, accounting for almost half of the whole flavor; the second was the fruity note, with a percentage of 31.39%. Both percentage contributions of the citrus and fruit notes totaled more than 70%, which were the main aroma in sweet orange juice; the floral, peely, fresh, woody, green and fatty notes accounted for 9.48%, 6.53%, 6.02%, 1.15%, 1.06% and 0.31%, respectively. Although they had a smaller percentage contribution of notes in the aroma of sweet orange, they were indispensable. They played roles in modifying the aroma of orange juice and made the aroma more full and rich. The establishment of the note contour of the sweet orange aroma can help flavorists to innovate and develop products related to sweet orange flavor, and help the fragrance companies and customers to quickly and efficiently evaluate and discuss sweet orange flavor in terms of its aroma.

### 2.4. Partial Least Squares Analysis Further Verifies the Relationship between Aroma Compounds and Notes

**Figure 2:**
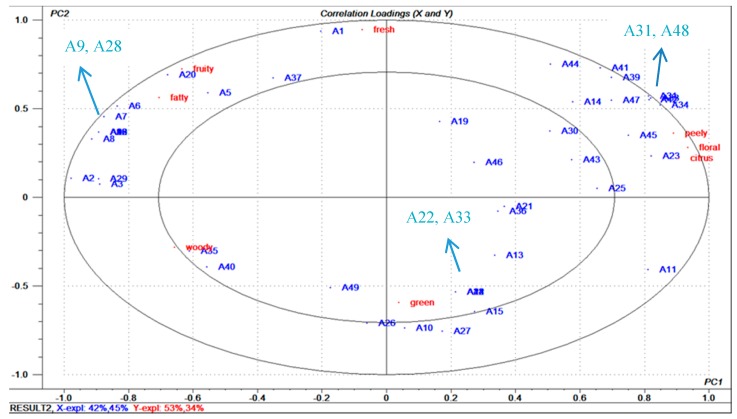
Analysis of correlation between aroma active substances and sensory properties in sweet orange juices (the compound name of A1~A49 is listed in Table 2).

As shown in Table 4, generic descriptive analysis and a ten-point line scale of sweet orange according to the aroma intensity were performed by six sensory panelists. Aromas of sweet orange were profiled with fresh, green, fruity, fatty, citrus, floral, peely and woody notes. Through the three replicates of sensory evaluation, obvious differences were found among the notes in the five sweet oranges. PLSR was applied to certify the correlation between the concentrations of aroma compounds detected by GC-MS and mean score of notes by sensory evaluation. The 49 odor-active compounds were used as X-matrix, and the 8 sensory notes were Y-matrix, which generated the correlation load diagram of PLSR, as shown in Figure 2.

From Figure 2, the blue dots are clustered near the red dots, indicating that these volatile compounds contributed to the note. Points within the circle indicate that the contribution of aroma was less than 50%, and points on the circle indicate that the contribution of the aroma was between 50% and 100%. The first quadrant basically distributes the fruity note and representative substances A7–A20 (including ethyl butyrate, ethyl 2-methylbutyrate, ethyl isovalerate), fresh note A1 (acetaldehyde) and fatty note A21,A28 (nonanal and octanal); the second quadrant distributes the peely note A31 (decanal), floral note A33, A38, A42, A47(linalool, hedion, neral, citral) and citrus note A11–A25 (α-pinene, 3-carene, myrcene, a-terpinene, limonene, 4-terpineol, o-Cymene,α-Terpinolene, 4-Isopropyltoluene), the scattered distribution is more intensive, and their aroma contribution is larger; the third quadrant is the woody note A35 (trans-caryophyllene); the fourth quadrant distributes the green note A10, A26, A27 (hexanol, hexanal, cis-3-hexen-1-ol). PLSR data analysis revalidates the high correlation between aroma substances detected by GC-O and notes of sensory evaluation.

### 2.5. Composition and Proportion of Sweet Orange Flavor

**Figure 3:**
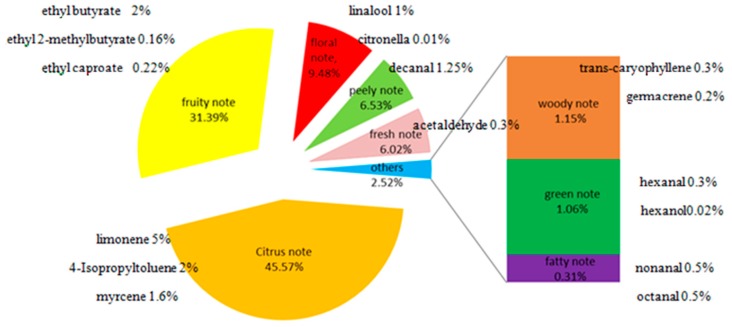
8 base modules of sweet orange flavor (the remaining proportion of each note comprises edible ethanol solvent).

On the basis of Table 3, representative volatile compounds with high OAVs were selected from each note. Based on the selection and matching of these characteristic substances with OAVs > 1, sweet orange flavor base modules were established according to the notes. The complex sweet orange flavor was split into a single note base module. Each note was composed of representative characteristic aroma substances and their usages. Through the sniffing training of the 8 base modules, different sweet orange flavors can be quickly evaluated (Figure 3). It could also be used by companies to communicate with customers or suppliers. Meanwhile, the sweet orange flavor can be optimized and innovated on the basis of the module by flavorists, and develop sweet orange tastes which are more popular with consumers.

## 3. Materials and Methods

### 3.1. Chemicals

An internal standard of 2-octanol (100 mg/L) and C4–C30 saturated alkanes (all chromatographically pure) was purchased from Sigma-Aldrich Co., Ltd. (St. Louis, MO, USA).

### 3.2. Materials

Sweet oranges from five different origins in China, including Zigui (ZG), Meishan (MS), Jiangyong (JY), Yaoxiang (YX) and Qingyang (QY) were selected for the experiment. ‘ZG’ were harvested from Hubei province, ‘MS’ were harvested Sichuan province, ‘JY’ were harvested from Hunan province, ‘YX’ were harvested from Guangxi province and ‘QY’ were harvested fromGansu province. These oranges were harvested at maturity period. The orange cores were removed and squeezed into juices by the JYL-C051 kitchen blender (Joyoung, Shandong, China). The filtered juices were kept in a refrigerator (−10 °C) until analyzed.

### 3.3. Solid Phase Microextraction (SPME) Adsorption of Aroma Compounds

10g orange juice was demulsified by adding 2 g NaCl and then quantitatively analyzed by adding 20 μL 2-octanol (100 mg/L) as internal standard. The juice was then placed in a 20 mL sealed solid microextraction vials and shaken evenly. Vials were kept at 45 °C in a water bath with 10 min of equilibration time. A SPME semi-quantification of volatile compounds was conducted according to some researches [23,24,25].

A 50/30 μm divinylbenzene/Carboxen/polydimethylsiloxane (DVB-CAR-PDMS) fiber (Supelco, Bellefonte, PA, USA) with a length of 1 cm was used. Extraction time was 45 min. Before chemical absorption, the fiber was preconditioned for 30 min on an Agilent 7890 gas chromatograph (Agilent Technologies, Santa Clara, CA, USA) with the injector temperature of 250 °C.

### 3.4. SPME-GC–MS of Volatile Compounds in Orange Juice

The volatile compounds were analyzed by a 7890A gas chromatograph with SPME and a 5973 mass selective detector (MSD) (Agilent Technologies). Samples were conducted using DB-Wax analytical fused silica capillary column (60 m × 0.25 mm × 0.25 μm; Agilent, Santa Clara, CA) and a DB-5 fused-silica capillary column (60 m × 0.25 mm × 0.25 μm, Agilent). Conditions for GC-MS analysis were as follows: the carrier gas was helium at a constant flow rate of 1 mL/min. The oven temperature was held at 50 °C, ramped at a rate of 10 °C/min to 100 °C for 5 min, and then ramped to 140 °C at a rate of 3 °C/min for 10 min, finally it reached 230 °C at a rate of 2 °C/min for 5 min. The desorption time was 5 min.

The MSD was used for chemical identification. Its electron ionization energy was 70 eV. The ion source temperature was set at 230 °C. The compounds were identified by matching retention time of authentic standards, retention indices (RIs), and mass spectra in the NIST 11 database. The RIs of unknown compounds were determined by alkanes C4–C30.

### 3.5. SPME-GC-FID-O Analysis of Orange Juice

GC-O analysis was conducted using an Agilent 7890A gas chromatograph equipped with a flame ionization detector (FID) and an ODP-2 olfactory port (Gerstel, Mulheim an der Ruhr, Germany). Separation of gas chromatographic was split into 1:1 (*v/v*) between flame ionization detector and sniffing. The purified and damp air flowing was transported to the olfactory assessor at a speed of 1.2 mL/min. Conditions for GC-O analysis and heating procedure referred to those of GC-MS.

The olfactory experiment was performed by 6 trained panelists (three females and three males, age: 22–40). Panelists were very sensitive to aroma identification due to olfactory training to reference compounds, sweet orange sample matrices in sniffing bottles, and experience in GC-O. Aroma characteristics and aroma intensity were recorded by the assessors based on 45 min of sniffing time. The intensity was calculated as the average of all panelists’ scores for identified aroma. The odor intensities were evaluated on a 10-point intensity line scale, where 0 meant a compound had a slight odor, 5 represented a moderate intensity and 10 was for an extremely strong sensation.

### 3.6. Sensory Evaluation

Based on the previous studies [26,27,28], the method of sensory analysis was generic descriptive analysis. 20 g orange juice was prepared in a 100 mL plastic cup with a Teflon cover for evaluation. At the beginning, the aroma of the orange juice was evaluated by a well-trained panel of 6 members (3 males and 3 females). Then, through the three preliminary consensus sessions (each 2 h), the panelists eventually reached a final agreement about the aroma description of the orange juice. Based on these discussions, the sweet orange juice was divided into eight notes: fresh, green, fruity, fatty, citrus, floral, peely and woody. A ten-point line scale, from 0 (not perceivable) to 3.0, 4.0, 5.0 (moderately perceivable), to 10.0 (very strongly perceivable), was given to the respective notes of orange juice according to the aroma intensity.

### 3.7. Odor Activity Values (OAV)

By using the formula of olfactory activity value, OAV = C/T, in which OAV represents the olfactory activity value of each flavor substance, C represents the concentration of the each compound, T represents the each compound of detection threshold in water. It is generally believed that aromatic compounds with high OAV are most likely to be the main contributors to the overall aroma. OAV > 1 indicates that the compounds have a direct impact on the aroma of sweet orange [29]. According to GC-O olfaction and the note classification of the sensory group, all volatile substances detected by GC-MS were classified into eight notes, and the OAV value of each note was the sum of the OAV values of all volatile substances in this note. Contribution percentage of a note in sweet orange juice = the OAV value of the note/the sum of all the OAV values of notes.

### 3.8. Statistical Analysis

In this study, relative standard deviation (RSD) better reflected the precision of GC-MS and GC-O tests data. Aroma intensity of GC-O and quantitative contents of volatile compounds were performed by analysis of variance (ANOVA). When there were significant differences between samples, Duncan’s multiple range tests were used with the level of significance set at *p* < 0.05. Both ANOVA and Duncan’s multiple range tests were conducted by PASW Statistics 18 (IBM, Chicago, IL, USA). Partial least squares regression (PLSR) was conducted by Unscrambler version 9.8 (CAMO ASA, Oslo, Norway). PLSR was used to further verify the relationship between flavor notes and corresponding aroma compounds.

## 4. Conclusions

Based on the results of GC-MS analysis and sensory evaluation, the percentage contribution of note OAVs in sweet orange were calculated and the proportion of aroma components in sweet orange could be visually displayed by the aroma contour diagram. By this method, the aroma quality of sweet orange flavor of the same type can be precisely and meticulously compared and evaluated, so as to develop more innovative and competitive orange flavor products. Meanwhile, the aroma module of orange flavor is conducive to communication between the fragrances and customers. A training system for sensory evaluation of orange flavor has been established to enable trainees to distinguish and identify the quality of sweet orange flavors from perspective of aroma.

## Figures and Tables

**Table 1:** Identification and quantification of volatile compounds in the sweet orange juices by GC-MS.

No.	RT	Compounds ^1^	RI ^2^		Identification ^3^	ZG	Concentration (μg/g) MS	JY	YX	QY
			DB-Wax	DB-5						
A1	4.479	acetaldehyde	690	396	MS, RI, Std	0.407 ± 0.03	-	-	-	0.336 ± 0.02
A2	5.174	ethyl acetate	887	610	MS, RI, Std	0.171 ± 0.009 c ^4^	0.178 ± 0.02 c	0.33 ± 0.027 b	0.17 ± 0.03 c	0.479 ± 0.05 a
A3	5.999	ethyl propionate	950	725	MS, RI, Std	- ^5^	0.0603 ± 0.008	0.0706 ± 0.008	-	0.136 ± 0.08
A4	6.108	ethyl isobutyrate	961	723	MS, RI, Std	-	-	-	-	0.078 ± 0.006
A5	6.417	methyl butyrate	984	729	MS, RI, Std	0.0982 ± 0.008 b	0.0482 ± 0.005 c	0.0898 ± 0.007 b	0.0534 ± 0.004 c	0.107 ± 0.03 a
A6	7.034	α-pinene	1018	940	MS, RI, Std	0.102 ± 0.02 b	0.0693 ± 0.005 c	0.0834 ± 0.009 c	0.0785 ± 0.006 c	0.193 ± 0.02 a
A7	7.234	ethyl butyrate	1040	808	MS, RI, Std	0.804 ± 0.07 b	0.106 ± 0.02 c	0.555 ± 0.04 b	0.113 ± 0.02 c	4.65 ± 0.54 a
A8	7.578	ethyl 2-methylbutyrate	1072	846	MS, RI, Std	-	0.0271 ± 0.003	0.0321 ± 0.004	-	0.371 ± 0.04
A9	7.816	ethyl isovalerate	1080	857	MS, RI, Std	-	-	-	-	0.05 ± 0.004
A10	8.148	hexanal	1082	801	MS, RI, Std	0.0545 ± 0.004 b	0.0513 ± 0.004 b	0.077 ± 0.006 a	0.066 ± 0.005 a	0.05 ± 0.003 b
A11	8.691	sabinene	1124	934	MS, RI, Std	0.0618 ± 0.005 b	0.0844 ± 0.006 a	0.0417 ± 0.005 b	0.104 ± 0.03 a	0.0107 ± 0.003 c
A12	8.818	camphene	1135	952	MS, RI, Std	-	-	-	0.0188 ± 0.003	-
A13	9.286	3-carene	1163	980	MS, RI, Std	0.0327 ± 0.004	-	0.0321 ± 0.002	0.0471 ± 0.005	0.00714 ± 0.0008
A14	9.602	myrcene	1167	991	MS, RI, Std	1.49 ± 0.13 a	0.609 ± 0.05 c	0.459 ± 0.05 c	1.06 ± 0.2 b	0.639 ± 0.05 c
A15	10.106	a-terpinene	1190	1017	MS, RI, Std	-	0.232 ± 0.03	0.135 ± 0.02	0.104 ± 0.03	0.025 ± 0.004
A16	10.244	propyl butyrate	1196	916	MS, RI, Std	-	-	-	-	0.0143 ± 0.002
A17	10.265	butanol	1146	660	MS, RI, Std	-	-	-	-	0.0107 ± 0.003
A18	11.099	limonene	1197	1027	MS, RI, Std	24.4 ± 1.92 a	19.2 ± 1.34 c	21.4 ± 2.19 b	22.8 ± 2.19 b	21.8 ± 2.37 b
A19	11.845	ethyl caproate	1235	999	MS, RI, Std	0.138 ± 0.02	0.0724 ± 0.005	-	-	0.361 ± 0.05
A20	11.963	γ-terpinene	1262	1062	MS, RI, Std	0.0691 ± 0.005 b	0.25 ± 0.03 a	0.0802 ± 0.009 b	0.0377 ± 0.005 c	0.05 ± 0.003 b
A21	11.98	octanal	1208	1020	MS, RI, Std	-	0.0213 ± 0.001	-	0.0061 ± 0.0005	0.022 ± 0.003
A22	12.019	α-phellandrene	1268	1027	MS, RI, Std	0.0145 ± 0.003	-	-	-	-
A23	12.842	o-cymene	1289	1025	MS, RI, Std	1.92 ± 0.23 b	2.46 ± 0.19 a	0.764 ± 0.06 c	0.848 ± 0.06 c	0.196 ± 0.02 d
A24	12.983	α-terpinolene	1295	1088	MS, RI, Std	-	-	-	0.132 ± 0.02	-
A25	13.288	4-isopropyltoluene	1280	1026	MS, RI, Std	1.85 ± 0.19 a	1.94 ± 0.23 a	1.84 ± 0.15 a	0.854 ± 0.07 b	0.614 ± 0.05 b
A26	14.792	hexanol	1357	858	MS, RI, Std		0.0091 ± 0.0008	0.0513 ± 0.004	0.0126 ± 0.002	-
A27	15.703	cis-3-hexen-1-ol	1388	857	MS, RI, Std	0.0109 ± 0.004	0.0151 ± 0.003	0.0513 ± 0.002	0.0251 ± 0.008	-
A28	16.035	nonanal	1396	1104	MS, RI, Std	-	-	-	-	0.00357 ± 0.0004
A29	16.958	ethyl caprylate	1436	1193	MS, RI, Std	0.00727 ± 0.0008	-	0.0128 ± 0.002	0.00942 ± 0.0004	0.0214 ± 0.003
A30	18.275	citronella	1488	1153	MS, RI, Std	0.0182 ± 0.002	0.006 ± 0.0008	0.0128 ± 0.004	-	-
A31	18.701	decanal	1502	1203	MS, RI, Std	0.0655 ± 0.007	0.0332 ± 0.004	-	0.022 ± 0.003	-
A32	19.26	ethyl 3-hydroxybutyrate	1551	1099	MS, RI, Std	0.00364 ± 0.0004	-	0.0032 ± 0.004	-	0.0214 ± 0.003
A33	19.748	linalool	1564	1069	MS, RI, Std	0.204 ± 0.03 a	0.109 ± 0.02 b	0.0449 ± 0.005 c	0.066 ± 0.003 c	0.0143 ± 0.009 d
A34	20.002	octanol	1604	1420	MS, RI, Std	0.0545 ± 0.006 a	0.0332 ± 0.004 b	0.0128 ± 0.003 c	0.0188 ± 0.007 c	0.00357 ± 0.0005 d
A35	21.335	trans-caryophyllene	1612	1179	MS, RI, Std	0.02184 ± 0.003	-	0.0257 ± 0.009	0.0188 ± 0.003	0.0107 ± 0.006
A36	21.398	4-terpineol	1672	1122	MS, RI, Std	-	0.0091 ± 0.0008	-	-	-
A37	23.145	ethyl 3-hydroxyhexanoate	1694	1241	MS, RI, Std	0.113 ± 0.04 a	0.0211 ± 0.007 c	0.0834 ± 0.004 b	0.0251 ± 0.003 c	0.134 ± 0.07 a
A38	23.313	neral	1704	1189	MS, RI, Std	0.0109 ± 0.004	0.006 ± 0.0007	0.0064 ± 0.0008	0.00628 ± 0.0009	-
A39	23.567	α-terpineol	1719	1495	MS, RI, Std	0.0291 ± 0.004	0.0181 ± 0.003	-	-	-
A40	24.212	valencene	1725	1555	MS, RI, Std	0.145 ± 0.04 c	0.0693 ± 0.007 d	0.728 ± 0.08 a	0.198 ± 0.03c	0.382 ± 0.05 b
A41	24.276	germacrene	1734	1247	MS, RI, Std	0.00727 ± 0.0009	0.003 ± 0.0004	-	-	-
A42	24.454	citral	1772	1228	MS, RI, Std	0.0327 ± 0.002	0.0151 ± 0.003	-	0.0126 ± 0.003	-
A43	24.927	citronellol	1860	1020	MS, RI, Std	0.00727 ± 0.0008	0.006 ± 0.0007	0.0064 ± 0.0009	-	-
A44	26.74	hexanoic acid	1894	1021	MS, RI, Std	0.00727 ± 0.0006	-	-	-	-
A45	31.102	octoic acid	2070	1186	MS, RI, Std	0.0109 ± 0.003	0.003 ± 0.0005	0.0032 ± 0.0004	0.00628 ± 0.0008	-
A46	33.127	pelargonic acid	2171	1267	MS, RI, Std	0.00364 ± 0.0005	-	0.0032 ± 0.0007	-	-
A47	35.593	hedion	2237	1673	MS, RI, Std	0.00364 ± 0.0005	0.003 ± 0.0002	-	-	-
A48	38.061	decanoic acid	2265	1374	MS, RI, Std	-	-	-	0.00314 ± 0.0005	-
A49	44.771	nootkatone	2563	1823	MS, RI, Std	-	-	0.0064 ± 0.0009	-	-

^1^ Volatile compounds detected in orange juice samples. ^2^ Retention index of compounds on DB-5 and DB-Wax columns. ^3^ MS: mass spectrum comparison using Wiley library. RI: retention index; Std: confirmed by the authentic standard. ^4^ Values with different roman letters (a–d) in the same row are significantly different according to the Duncan test (*p* < 0.05). ^5^ not detected.

**Table 2:** Aroma compounds of five sweet oranges by GC-O analysis with aroma description and aroma intensity.

No.	Compounds ^1^	RI ^2^		Identification ^3^	Aroma Description	Aroma Intensity ^4^									
		DB-Wax	DB-5			ZG	RSD (%)	MS	RSD (%)	JY	RSD (%)	YX	RSD (%)	QY	RSD (%)
A1	acetaldehyde	690	396	AD, RI, Std	fresh, aldehydic	5.9	6.8	-	-	-	-	-	-	5.4	7.3
A2	ethyl acetate	887	610	AD, RI, Std	pear, fruity	2.8c	8.7	3.0c	7.1	3.7b	8.4	3.9b	6.7	4.5a	6.8
A3	ethyl propionate	950	725	AD, RI, Std	wine, fruity	- ^5^	-	1.3	23.1	1.5	12.9	-	-	2.6	8.9
A4	ethyl isobutyrate	961	723	AD, RI, Std	pineapple	-	-	-	-	-	-	-	-	1.7	13.0
A5	methyl butyrate	984	729	AD, RI, Std	fruity	2.9a	9.5	2.4a	5.9	2.8a	8.5	2.5a	11.2	2.3a	10.7
A6	α-pinene	1018	940	AD, RI, Std	woody	2.5a	8.9	1.6b	13.8	1.8b	13.1	1.7b	9.8	2.8a	11.4
A7	ethyl butyrate	1040	808	AD, RI, Std	pineapple	6.6b	7.9	4.2c	5.3	5.3b	9.4	3.2c	7.3	7.5a	3.6
A8	ethyl 2-methylbutyrate	1072	846	AD, RI, Std	pineapple	-	-	0.2	53.6	0.3	42.0	-	-	1.3	26.2
A9	ethyl isovalerate	1080	857	AD, RI, Std	banana	-	-	-	-	-	-	-	-	2.5	9.9
A10	hexanal	1082	801	AD, RI, Std	green, fatty	3.9a	3.8	3.6a	7.5	2.7b	9.1	2.6b	8.2	2.5b	7.3
A11	sabinene	1124	934	AD, RI, Std	citrus, woody	3.6a	6.4	3.8a	6.8	3.4a	5.6	2.3b	8.3	1.2c	13.2
A12	camphene	1135	952	AD, RI, Std	woody	-	-	-	-	-	-	0.3	42.0	-	-
A13	3-carene	1163	980	AD, RI, Std	citrus	1.3	4.8	-	-	1.9	13.1	1.4	13.7	1.2	16.3
A14	myrcene	1167	991	AD, RI, Std	floral	6.2a	2.2	5.3b	4.8	5.2b	6.8	6.3a	6.7	1.3c	11.2
A15	a-terpinene	1190	1017	AD, RI, Std	woody, spearmint	-	-	5.10	3.8	4.9	7.3	2.1	9.1	1.7	12.4
A16	propyl butyrate	1196	916	AD, RI, Std	wine, fruity	-	-	-	-	-	-	-	-	1.9	12.1
A17	butanol	1146	660	AD, RI, Std	wine	-	-	-	-	-	-	-	-	2.3	10.1
A18	limonene	1197	1027	AD, RI, Std	lemon	7.8a	7.4	7.5a	6.4	7.6a	8.3	7.7a	4.9	7.6a	6.8
A19	ethyl caproater	1235	999	AD, RI, Std	fruity	3.9	7.8	2.7	4.9	-	-	-	-	4.3	5.9
A20	γ-terpinene	1262	1062	AD, RI, Std	woody, spearmint	0.7b	20.2	1.5a	7.3	0.8b	24.9	0.3c	52.0	0.5c	32.3
A21	octanal	1208	1020	AD, RI, Std	spicy, herbal	-	-	1.3	16.1	-	-	1.5	12.9	1.8	13.9
A22	α-phellandrene	1268	1027	AD, RI, Std	spicy, herbal	2.2	5.7	-	-	-	-	-	-	-	-
A23	o-cymene	1289	1025	AD, RI, Std	woody, herbal	4.1a	5.5	4.2a	4.5	2.7b	4.6	2.8b	7.8	1.2c	14.9
A24	α-terpinolene	1295	1088	AD, RI, Std	spicy, herbal	-	-	-	-	-	-	1.3	16.1	-	
A25	4-isopropyltoluene	1280	1026	AD, RI, Std	green, leave	7.5a	9.9	7.4a	8.9	7.2a	5.4	3.8b	6.9	1.6c	14.8
A26	hexanol	1357	858	AD, RI, Std	irritation, stink			1.9	19.6	2.2	8.1	1.8	10.7	-	-
A27	cis-3-hexen-1-ol	1388	857	AD, RI, Std	fatty, grassy, leaves	2.3	6.7	2.2	5.7	1.9	13.8	3.8	7.3	-	-
A28	nonanal	1396	1104	AD, RI, Std	floral, citrus	-	-	-	-	-	-	-	-	1.2	21.8
A29	ethyl caprylate	1436	1193	AD, RI, Std	fruity	2.8	8.9	-	-	2.7	6.7	2.6	10.0	3.3	6.7
A30	citronella	1488	1153	AD, RI, Std	rosy	2.4	7.4	1.6	20.9	1.5	15.3	-	-	-	-
A31	decanal	1502	1203	AD, RI, Std	fatty	4.2	6.3	3.3	7.1	-	-	2.9	10.1	-	-
A32	ethyl 3-hydroxybutyrate	1551	1099	AD, RI, Std	fruity	0.7	20.5	-	-	1.7	8.7	-	-	2.1	28.9
A33	linalool	1564	1069	AD, RI, Std	floral, woody	7.2a	2.6	3.9c	5.8	3.1c	8.5	4.6b	5.7	1.7a	16.6
A34	octanol	1604	1420	AD, RI, Std	irritation, stink	1.5b	12.9	1.7a	17.5	1.3b	15.0	1.4b	16.9	0.3c	41.3
A35	trans-caryophyllene	1612	1179	AD, RI, Std	spicy	1.2	23.5	-	-	1.4	18.0	1.3	4.5	1.4	17.5
A36	4-terpineol	1672	1122	AD, RI, Std	woody, floral	-	-	1.2	18.5	-	-	-	-	-	-
A37	ethyl 3-hydroxyhexanoate	1694	1241	AD, RI, Std	fruity	2.6b	9.5	2.5b	7.6	2.7b	8.5	2.3b	10.1	3.4a	4.6
A38	neral	1704	1189	AD, RI, Std	lemon	3.5	3.7	2.6	7.2	2.6	10.7	2.8	12.9	-	-
A39	α-terpineol	1719	1495	AD, RI, Std	woody, spearmint	1.7	23.9	1.8	10.7	-	-	-	-	-	-
A40	valencene	1725	1555	AD, RI, Std	fruity	3.4b	5.6	3.7b	9.6	3.8b	5.4	4.4a	6.6	4.9a	5.8
A41	germacrene	1734	1247	AD, RI, Std	earthy	1.9	16.9	1.7	14.1	-	-	-	-	-	-
A42	citral	1772	1228	AD, RI, Std	lemon, aldehyde	2.3	7.5	2.2	10.8	-	-	2.6	9.5	-	-
A43	citronellol	1860	1020	AD, RI, Std	rosy, sweet	3.7	8.9	3.6	6.9	3.5	7.7	-	-	-	-
A44	hexanoic acid	1894	1021	AD, RI, Std	rancid flavor	1.7	13.9	-	-	-	-	-	-	-	-
A45	octoic acid	2070	1186	AD, RI, Std	weak milk, fatty	1.9	13.2	1.3	16.2	1.2	9.8	0.6	25.2	-	-
A46	pelargonic acid	2171	1267	AD, RI, Std	weak milk, fatty	2.1	6.4	-	-	1.3	8.4	-	-	-	-
A47	hedion	2237	1673	AD, RI, Std	floral, jasmine	0.8	29.1	0.9	28.8	-	-	-	-	-	-
A48	decic acid	2265	1374	AD, RI, Std	weak milk, fatty	-	-	-	-	-	-	1.3	12.2	-	-
A49	nootkatone	2563	1823	AD, RI, Std	fruity, citrus	-	-	-	-	2.6	5.7	-	-	-	-

^1^ Volatile compounds detected in sweet orange juice samples. ^2^ Retention index of compounds on DB-5 and DB-Wax columns. ^3^ RI: retention index; Std: confirmed by the authentic standard; AD: Aroma descriptor. ^4^ Values with different roman letters (a–c) in the same row are significantly different according to the Duncan test (*p* < 0.05). ^5^ not detected.

**Table 3:** Average OAV value and aroma note percentage of volatile compounds in sweet orange.

No.	Compounds ^A^	Thresholds (μg/g)	Literatures ^B^	OAV (C/T) ^C^					Note OAV	Note OAV% ^E^
				ZG	MS	JY	YX	QY		
1	acetaldehyde	0.01	1	41	- ^D^	-	-	34	fresh 37.15	6.02
2	hexanal	0.0091	2	6	6	8	7	5	green	
3	hexanol	0.161	2	-	<1	1	1	-	6.51	1.06
4	ethyl propionate	0.1	3	-	<1	<1	-	1	fruity 193.56	31.39
5	ethyl isobutyrate	0.015	3	-	-	-	-	5		
6	ethyl butyrate	0.02	3	40	5	28	6	233		
7	ethyl 2-methylbutyrate	0.002	3	-	14	16	-	186		
8	ethyl isovalerate	0.006	3	-	-	-	-	8		
9	ethyl caproate	0.005	3	28	14	-	-	72		
10	nonanal	0.015	2	-	-	-	-	1	fatty 1.804	0.31
11	octanal	0.012	2	-	2	-	<1	2		
12	α-pinene	0.19	2	<1	<1	<1	<1	1	citrus 280.31	45.47
13	3-carene	0.044	2	<1	-	<1	1	<1		
14	myrcene	0.0166	2	90	37	28	64	38		
15	a-terpinene	0.085	2	-	3	2	1	<1		
16	limonene	0.2	2	122	96	107	114	109		
17	4-terpineol	0.005	2	-	2	-	-	-		
18	o-Cymene	0.4	2	5	6	2	2	<1		
19	α-Terpinolene	0.041	2	-	-	-	3	-		
20	4-Isopropyltoluene	0.0133	2	139	146	138	64	46		
21	linalool	0.0015	2	136	73	30	44	10	floral 58.42	9.48
22	hedion	5.7	2	1	1	-	-	-		
23	decanal	0.001	4	66	33	-	22	-	peel 40.23	6.53
24	trans-caryophyllene	0.06	5	2	-	12	3	6	woody 7.07	1.15
25	germacrene	0.016	5	1	1	-	-	-		

Numbers 1–5 listed in the Literature column mean that thresholds of the compounds were detected by: 1, Milo and Grosch (1997); 2, Tamura et al. (2001); 3, Schnabel et al. (1988); 4, Padrayuttawat et al. (1997); 5, Buttery et al. (1987), according to ref [22]. ^A^ The volatile compounds with OAVs ≥ 1. ^B^ The odor threshold (μg/g) calculated in water referred to in the literature. ^C^ The OAV of each volatile compound. ^D^ not detected. ^E^ Percentage contribution of notes in sweet orange.

**Table 4:** The mean intensity values of the eight attributes for the five sweet oranges in descriptive sensory evaluation.

Sample	Mean Score							
	Fresh	Green	Fruity	Fatty	Citrus	Floral	Peely	Woody
O1	3.5 c	3 c	5.4 bc	0.3 d	9.8 a	6.5 b	6.1 b	2 cd
O2	0 c	2.6 bc	3.6 b	0 c	9.2 a	4.8 b	5 b	1 c
O3	0 c	4.7 b	4 b	0 c	9 a	3.9 b	0.3 c	4.2 b
O4	0 d	3.3 b	1 cd	0.2 d	8.8 a	4.2 b	3.6 b	2.4 c
O5	2.3 b	2.5 b	8 a	0.8 c	8 a	2 b	0.2 c	3.3 b

Values with different roman letters (a–d) in the same row are significantly different according to the Duncan test (*p* < 0.05).
